# Supplementary material for: Revisiting the guidelines for ending isolation for COVID-19 patients
Source: eLife. 2021 Jul 27;10:e69340. doi: 10.7554/eLife.69340 (PMC8315804; doi:10.7554/eLife.69340)
Supplement: Figure 1—source data 1. — The numbers in parentheses are the 95% empirical CI. [file elife-69340-fig1-data1.docx]

**Figure 1-source data 1. Estimated viral load curves from the three analyzed models**

| Day after symptom onsets | Viral load (log_10_ RNA copies/ml) | | |
| --- | --- | --- | --- |
|  | Baseline model | “Eclipse phase” model | “Innate immune response” model |
| 0 | 4.514  (3.936 to 4.722) | 4.719  (4.230 to 4.811) | 4.588  (4.324 to 4.591) |
| 1 | 5.409  (4.625 to 6.159) | 5.536  (4.931 to 6.216) | 5.465  (3.861 to 6.925) |
| 2 | 5.348  (4.918 to 5.879) | 5.652  (5.007 to 6.004) | 5.883  (3.672 to 6.726) |
| 3 | 5.091  (4.696 to 5.630) | 5.412  (4.754 to 5.725) | 5.582  (3.576 to 6.457) |
| 4 | 4.810  (4.419 to 5.392) | 5.079  (4.302 to 5.472) | 5.113  (2.805 to 6.080) |
| 5 | 4.524  (4.124 to 5.154) | 4.726  (3.848 to 5.201) | 4.644  (1.777 to 5.643) |
| 6 | 4.236  (3.789 to 4.916) | 4.367  (3.393 to 4.922) | 4.210  (0.755 to 5.263) |
| 7 | 3.947  (3.405 to 4.678) | 4.008  (2.939 to 4.709) | 3.809  (-0.262 to 5.196) |
| 8 | 3.658  (3.022 to 4.441) | 3.647  (2.484 to 4.518) | 3.433  (-1.277 to 4.980) |
| 9 | 3.369  (2.638 to 4.203) | 3.286  (2.029 to 4.317) | 3.070  (-2.291 to 4.701) |
| 10 | 3.080  (2.254 to 3.965) | 2.926  (1.575 to 4.251) | 2.717  (-3.305 to 4.617) |
| 11 | 2.790  (1.870 to 3.727) | 2.565  (1.120 to 4.121) | 2.368  (-4.318 to 4.644) |
| 12 | 2.501  (1.487 to 3.489) | 2.204  (0.665 to 4.051) | 2.021  (-5.332 to 4.635) |
| 13 | 2.212  (1.103 to 3.251) | 1.843  (0.211to 3.916) | 1.676  (-6.345 to 4.587) |
| 14 | 1.922  (0.715 to 3.013) | 1.482  (0.050 to 3.803) | 1.331  (-7.358 to 4.506) |
| 15 | 1.633  (0.322 to 2.775) | 1.121  (-0.399 to 3.750) | 0.987  (-8.371 to 4.400) |
| 16 | 1.344  (-0.027 to 2.537) | 0.761  (-0.848 to 3.622) | 0.642  (-9.384 to 4.277) |
| 17 | 1.054  (-0.331 to 2.299) | 0.400  (-1.297 to 3.475) | 0.298  (-10.397 to 4.152) |
| 18 | 0.765  (-0.710 to 2.061) | 0.039  (-1.746 to 3.340) | -0.046  (-11.411 to 4.051) |
| 19 | 0.476  (-1.089 to 1.823) | -0.322  (-2.195 to 3.209) | -0.390  (-12.424 to 3.943) |
| 20 | 0.186  (-1.468 to 1.585) | -0.683  (-2.645 to 3.101) | -0.734  (-13.437 to 3.831) |

Note: The numbers in parentheses are the 95% empirical CI.
