# Supplementary material for: Revisiting the guidelines for ending isolation for COVID-19 patients
Source: eLife. 2021 Jul 27;10:e69340. doi: 10.7554/eLife.69340 (PMC8315804; doi:10.7554/eLife.69340)
Supplement: Figure 2—source data 1. — The numbers in parentheses are the 95% empirical CI. [file elife-69340-fig2-data1.docx]

**Figure 2-source data 1. Probability of prematurely ending isolation and mean length of unnecessarily prolonged isolation under the one-size-fit-all approach**

|  | Probability of prematurely ending isolation | | | Mean length of unnecessarily prolonged isolation (days) | | |
| --- | --- | --- | --- | --- | --- | --- |
| isolation period  (days) | Infectiousness threshold values | | | Infectiousness threshold values | | |
|  | 10^4.5^ copies/mL | 10^5.0^ copies/mL | 10^5.5^ copies/mL | 10^4.5^ copies/mL | 10^5.0^ copies/mL | 10^5.5^ copies/mL |
| 1 | 0.997  (0.995 to 0.999) | 0.981  (0.976 to 0.985) | 0.943  (0.930 to 0.956) | -4.2  (-12 to -2) | -2.2  (-8 to -1) | -0.7  (-4 to 0) |
| 2 | 0.990  (0.987 to 0.993) | 0.941  (0.933 to 0.948) | 0.568  (0.540 to 0.596) | -3.2  (-11 to -1) | -1.2  (-7 to 0) | 0.3  (-3 to 1) |
| 3 | 0.928  (0.919 to 0.936) | 0.620  (0.604 to 0.636) | 0.145  (0.125 to 0.165) | -2.2  (-10 to 0) | -0.2  (-6 to 1) | 1.3  (-2 to 2) |
| 4 | 0.728  (0.714 to 0.742) | 0.332  (0.316 to 0.347) | 0.060  (0.047 to 0.073) | -1.2  (-9 to 1) | 0.8  (-5 to 2) | 2.3  (-1 to 3) |
| 5 | 0.511  (0.495 to 0.526) | 0.161  (0.149 to 0.173) | 0.013  (0.006 to 0.019) | -0.2  (-8 to 2) | 1.8  (-4 to 3) | 3.3  (0 to 4) |
| 6 | 0.343  (0.328 to 0.358) | 0.084  (0.075 to 0.093) | 0 | 0.8  (-7 to 3) | 2.8  (-3 to 4) | 4.3  (1 to 5) |
| 7 | 0.220  (0.207 to 0.233) | 0.050  (0.042 to 0.057) | 0 | 1.8  (-6 to 4) | 3.8  (-2 to 5) | 5.3  (2 to 6) |
| 8 | 0.151  (0.139 to 0.162) | 0.029  (0.024 to 0.035) | 0 | 2.8  (-5 to 5) | 4.8  (-1 to 6) | 6.3  (3 to 7) |
| 9 | 0.092  (0.083 to 0.102) | 0.020  (0.016 to 0.025) | 0 | 3.8  (-4 to 6) | 5.8  (0 to 7) | 7.3  (4 to 8) |
| 10 | 0.066  (0.058 to 0.074) | 0.009  (0.006 to 0.012) | 0 | 4.8  (-3 to 7) | 6.8  (1 to 8) | 8.3  (5 to 9) |
| 11 | 0.048  (0.041 to 0.055) | 0.002  (0.001 to 0.004) | 0 | 5.8  (-2 to 8) | 7.8  (2 to 9) | 9.3  (6 to 10) |
| 12 | 0.034  (0.028 to 0.040) | 0 | 0 | 6.8  (-1 to 9) | 8.8  (3 to 10) | 10.3  (7 to 11) |
| 13 | 0.024  (0.019 to 0.029) | 0 | 0 | 7.8  (0 to 10) | 9.8  (4 to 11) | 11.3  (8 to 12) |
| 14 | 0.017  (0.013 to 0.021) | 0 | 0 | 8.8  (1 to 11) | 10.8  (5 to 12) | 12.3  (9 to 13) |
| 15 | 0.012  (0.009 to 0.016) | 0 | 0 | 9.8  (2 to 12) | 11.8  (6 to 13) | 13.3  (10 to 14) |
| 16 | 0.008  (0.005 to 0.011) | 0 | 0 | 10.8  (3 to 13) | 12.8  (7 to 14) | 14.3  (11 to 15) |
| 17 | 0.003  (0.001 to 0.005) | 0 | 0 | 11.8  (4 to 14) | 13.8  (8 to 15) | 15.3  (12 to 16) |
| 18 | 0.002  (0.001 to 0.003) | 0 | 0 | 12.8  (5 to 15) | 14.8  (9 to 16) | 16.3  (13 to 17) |
| 19 | 0.001  (0 to 0.002) | 0 | 0 | 13.8  (6 to 16) | 15.8  (10 to 17) | 17.3  (14 to 18) |
| 20 | 0 | 0 | 0 | 14.8  (7 to 17) | 16.8  (11 to 18) | 18.3  (15 to 19) |

Note: The numbers in parentheses are the 95% empirical CI.
