# Supplementary material for: Revisiting the guidelines for ending isolation for COVID-19 patients
Source: eLife. 2021 Jul 27;10:e69340. doi: 10.7554/eLife.69340 (PMC8315804; doi:10.7554/eLife.69340)
Supplement: Figure 3—source data 1. — The cell with numbers in bold corresponds to the baseline. The numbers in parentheses are the 95% CI. [file elife-69340-fig3-data1.docx]

Figure 3-source data 1. Probability of prematurely ending isolation of infectious patients with different guidelines (with $\boldsymbol{10}^{\boldsymbol{5.0}}$ copies/mL as an infectiousness threshold value)

|  |  | Interval of tests | | | | |
| --- | --- | --- | --- | --- | --- | --- |
|  |  | 1 day | 2 days | 3 days | 4 days | 5 days |
| Consecutive negative results | 1 | 0.393  (0.377 to 0.409) | 0.245  (0.231 to 0.259) | 0.181  (0.168 to 0.194) | 0.137  (0.126 to 0.148) | 0.102  (0.092 to 0.112) |
|  | 2 | **0.081**  **(0.072 to 0.090)** | 0.066  (0.058 to 0.074) | 0.142  (0.131 to 0.154) | 0.022  (0.017 to 0.026) | 0.008  (0.005 to 0.011) |
|  | 3 | 0.020  (0.016 to 0.025) | 0.001  (0 to 0.002) | 0.002  (0.001 to 0.004) | 0 | 0 |
|  | 4 | 0 | 0 | 0 | 0 | 0 |
|  | 5 | 0 | 0 | 0 | 0 | 0 |

Note: The cell with numbers in bold corresponds to the baseline. The numbers in parentheses are the 95%CI.
