# Supplementary material for: Revisiting the guidelines for ending isolation for COVID-19 patients
Source: eLife. 2021 Jul 27;10:e69340. doi: 10.7554/eLife.69340 (PMC8315804; doi:10.7554/eLife.69340)
Supplement: Figure 3—source data 2. — The cell with numbers in bold corresponds to the baseline. The numbers in parentheses are the empirical 95% CI. [file elife-69340-fig3-data2.docx]

Figure 3-source data 2. Length of unnecessarily prolonged isolation with different guidelines (with $\boldsymbol{10}^{\boldsymbol{5.0}}$ copies/mL as an infectiousness threshold value)

|  |  | Interval of tests | | | | |
| --- | --- | --- | --- | --- | --- | --- |
|  |  | 1 day | 2 days | 3 days | 4 days | 5 days |
| Consecutive negative results | 1 | -0.4  (-3 to 1) | 0.4  (-3 to 3) | 0.8  (-2 to 4) | 1.6  (-2 to 5) | 2.1  (-2 to 6) |
|  | 2 | **1.2**  **(-1 to 3)** | 2.3  (-1 to 5) | 2.8  (-2 to 7) | 5.0  (0 to 9) | 6.2  (1 to 11) |
|  | 3 | 2.3  (0 to 5) | 4.5  (1 to 8) | 5.9  (1 to 10) | 9.0  (4 to 13) | 11.2  (6 to 16) |
|  | 4 | 3.4  (1 to 6) | 6.5  (3 to 10) | 8.9  (4 to 13) | 13.0  (8 to 17) | 16.2  (11 to 21) |
|  | 5 | 4.5  (2 to 8) | 8.5  (5 to 12) | 11.9  (7 to 16) | 17.0  (12 to 21) | 21.2  (16 to 26) |

Note: The cell with numbers in bold corresponds to the baseline. The numbers in parentheses are the empirical 95%CI.
