# Supplementary material for: Revisiting the guidelines for ending isolation for COVID-19 patients
Source: eLife. 2021 Jul 27;10:e69340. doi: 10.7554/eLife.69340 (PMC8315804; doi:10.7554/eLife.69340)
Supplement: Figure 3—source data 3. — The cell with numbers in bold corresponds to the baseline. The numbers in parentheses are the 95% CI. [file elife-69340-fig3-data3.docx]

Figure 3-source data 3. Probability of prematurely ending isolation of infectious patients with different guidelines (with $\boldsymbol{10}^{\boldsymbol{4.5}}$ copies/mL as an infectiousness threshold value)

|  |  | Interval of tests | | | | |
| --- | --- | --- | --- | --- | --- | --- |
|  |  | 1 day | 2 days | 3 days | 4 days | 5 days |
| Consecutive negative results | 1 | 0.447  (0.431 to 0.463) | 0.254  (0.240 to 0.268) | 0.177  (0.165 to 0.189) | 0.140  (0.129 to 0.151) | 0.108  (0.098 to 0.117) |
|  | 2 | **0.065**  **(0.057 to 0.073)** | 0.019  (0.015 to 0.023) | 0.036  (0.030 to 0.042) | 0.009  (0.006 to 0.012) | 0.009  (0.006 to 0.012) |
|  | 3 | 0.011  (0.008 to 0.014) | 0.001  (0 to 0.002) | 0. | 0 | 0 |
|  | 4 | 0 | 0 | 0 | 0 | 0 |
|  | 5 | 0 | 0 | 0 | 0 | 0 |

Note: The cell with numbers in bold corresponds to the baseline. The numbers in parentheses are the 95%CI.
