# Supplementary material for: Revisiting the guidelines for ending isolation for COVID-19 patients
Source: eLife. 2021 Jul 27;10:e69340. doi: 10.7554/eLife.69340 (PMC8315804; doi:10.7554/eLife.69340)
Supplement: Figure 3—source data 4. — The cell with numbers in bold corresponds to the baseline. The numbers in parentheses are the empirical 95% CI. [file elife-69340-fig3-data4.docx]

Figure 3-source data 4. Length of unnecessarily prolonged isolation with different guidelines (with $\boldsymbol{10}^{\boldsymbol{4.5}}$ copies/mL as an infectiousness threshold value)

|  |  | Interval of tests | | | | |
| --- | --- | --- | --- | --- | --- | --- |
|  |  | 1 day | 2 days | 3 days | 4 days | 5 days |
| Consecutive negative results | 1 | -0.4  (-4 to 2) | 0.3  (-3 to 3) | 1.0  (-2 to 4) | 1.4  (-2 to 5) | 2.0  (-2 to 6) |
|  | 2 | **1.2**  **(-1 to 3)** | 2.6  (0 to 5) | 3.9  (-1 to 7) | 5.5  (0 to 9) | 6.9  (1 to 11) |
|  | 3 | 2.3  (0 to 5) | 4.7  (2 to 7) | 7.0  (2 to 10) | 9.5  (4 to 13) | 11.9  (6 to 16) |
|  | 4 | 3.4  (1 to 6) | 6.7  (4 to 10) | 10.0  (5 to 13) | 13.5  (8 to 17) | 16.9  (11 to 21) |
|  | 5 | 4.5  (2 to 7) | 8.7  (6 to 12) | 13.0  (8 to 16) | 17.5  (12 to 21) | 21.9  (16 to 26) |

Note: The cell with numbers in bold corresponds to the baseline. The numbers in parentheses are the empirical 95%CI.
