# Supplementary material for: Revisiting the guidelines for ending isolation for COVID-19 patients
Source: eLife. 2021 Jul 27;10:e69340. doi: 10.7554/eLife.69340 (PMC8315804; doi:10.7554/eLife.69340)
Supplement: Figure 3—source data 5. — The cell with numbers in bold corresponds to the baseline. The numbers in parentheses are the 95% CI. [file elife-69340-fig3-data5.docx]

Figure 3-source data 5. Probability of prematurely ending isolation of infectious patients with different guidelines (with $\boldsymbol{10}^{\boldsymbol{5.5}}$ copies/mL as an infectiousness threshold value)

|  |  | Interval of tests | | | | |
| --- | --- | --- | --- | --- | --- | --- |
|  |  | 1 day | 2 days | 3 days | 4 days | 5 days |
| Consecutive negative results | 1 | 0.215  (0.191 to 0.238) | 0.110  (0.093 to 0.128) | 0.054  (0.041 to 0.066) | 0.063  (0.049 to 0.077) | 0.047  (0.035 to 0.059) |
|  | 2 | **0.051**  **(0.038 to 0.063)** | 0.101  (0.084 to 0.118) | 0.066  (0.052 to 0.080) | 0 | 0 |
|  | 3 | 0.035  (0.024 to 0.045) | 0.006  (0.002 to 0.011) | 0 | 0 | 0 |
|  | 4 | 0.009  (0.004 to 0.015) | 0 | 0 | 0 | 0 |
|  | 5 | 0.006  (0.002 to 0.011) | 0 | 0 | 0 | 0 |

Note: The cell with numbers in bold corresponds to the baseline. The numbers in parentheses are the 95%CI.
