# Supplementary material for: Revisiting the guidelines for ending isolation for COVID-19 patients
Source: eLife. 2021 Jul 27;10:e69340. doi: 10.7554/eLife.69340 (PMC8315804; doi:10.7554/eLife.69340)
Supplement: Figure 3—source data 6. — The cell with numbers in bold corresponds to the baseline. The numbers in parentheses are the empirical 95% CI. [file elife-69340-fig3-data6.docx]

Figure 3-source data 6. Length of unnecessarily prolonged isolation with different guidelines (with $\boldsymbol{10}^{\boldsymbol{5.5}}$ copies/mL as an infectiousness threshold value)

|  |  | Interval of tests | | | | |
| --- | --- | --- | --- | --- | --- | --- |
|  |  | 1 day | 2 days | 3 days | 4 days | 5 days |
| Consecutive negative results | 1 | 0.1  (-2 to 2) | 0.8  (-1 to 3) | 1.3  (-1 to 4) | 1.9  (-1 to 5) | 2.4  (-1 to 6) |
|  | 2 | **1.2**  **(-1 to 4)** | 2.3  (-1 to 6) | 1.9  (-2 to 7) | 4.7  (1 to 9) | 5.9  (1 to 11) |
|  | 3 | 2.2  (-1 to 5) | 4.5  (1 to 8) | 5.0  (2 to 10) | 8.7  (5 to 14) | 10.9  (6 to 16) |
|  | 4 | 3.4  (0 to 6) | 6.5  (3 to 10) | 8.0  (5 to 13) | 12.7  (9 to 18) | 15.9  (11 to 21) |
|  | 5 | 4.5  (1 to 7) | 8.6  (5 to 12) | 11.0  (8 to 16) | 16.7  (13 to 22) | 20.9  (16 to 26) |

Note: The cell with numbers in bold corresponds to the baseline. The numbers in parentheses are the empirical 95%CI.
