# Supplementary material for: Revisiting the guidelines for ending isolation for COVID-19 patients
Source: eLife. 2021 Jul 27;10:e69340. doi: 10.7554/eLife.69340 (PMC8315804; doi:10.7554/eLife.69340)
Supplement: Figure 5—source data 1. — The numbers in parentheses are the empirical 95% CI. [file elife-69340-fig5-data1.docx]

Figure 5-source data 1. Mean length of unnecessarily prolonged isolation (days) with different guidelines and infectiousness threshold values controlling the risk of prematurely ending isolation ≤ 5% and ≤ 1% for the three analyzed models

| Baseline model | Risk of prematurely ending isolation ≤ 5% | Infectiousness threshold values (copies/mL) | | |
| --- | --- | --- | --- | --- |
|  |  | 10^4.5^ | 10^5.0^ | 10^5.5^ |
|  | One-size-fits-all approach | 5.8 (-2 to 8) | 3.8 (-2 to 5) | 3.3 (0 to 4) |
|  | Personalized approach | 2.3 (0 to 5) | 2.3 (0 to 5) | 2.4 (-1 to 6) |
|  | Risk of prematurely ending isolation ≤ 1% | Infectiousness threshold values (copies/mL) | | |
|  |  | 10^4.5^ | 10^5.0^ | 10^5.5^ |
|  | One-size-fits-all approach | 10.8 (3 to 13) | 6.8 (1 to 8) | 4.3 (1 to 5) |
|  | Personalized approach | 3.4 (1 to 6) | 3.4 (1 to 6) | 3.4 (0 to 6) |
| “Eclipse phase” model | Risk of prematurely ending isolation ≤ 5% | Infectiousness threshold values (copies/mL) | | |
|  |  | 10^4.5^ | 10^5.0^ | 10^5.5^ |
|  | One-size-fits-all approach | 5.9 (0 to 10) | 4.3 (0 to 7) | 3.5 (2 to 6) |
|  | Personalized approach | 1.1 (-1 to 3) | 2.2 (0 to 4) | 1.2 (-1 to 3) |
|  | Risk of prematurely ending isolation ≤ 1% | Infectiousness threshold values (copies/mL) | | |
|  |  | 10^4.5^ | 10^5.0^ | 10^5.5^ |
|  | One-size-fits-all approach | 9.9 (5 to 15) | 6.3 (3 to 10) | 4.5 (3 to 7) |
|  | Personalized approach | 2.2 (0 to 5) | 3.3 (1 to 6) | 3.2 (0 to 5) |
| “Innate immune response” model | Risk of prematurely ending isolation ≤ 5% | Infectiousness threshold values (copies/mL) | | |
|  |  | 10^4.5^ | 10^5.0^ | 10^5.5^ |
|  | One-size-fits-all approach | 6.6 (0 to 12) | 5.5 (2 to 8) | 4.5 (2 to 6) |
|  | Personalized approach | 1.1 (0 to 3) | 1.1 (0 to 3) | 1.0 (0 to 2) |
|  | Risk of prematurely ending isolation ≤ 1% | Infectiousness threshold values (copies/mL) | | |
|  |  | 10^4.5^ | 10^5.0^ | 10^5.5^ |
|  | One-size-fits-all approach | 13.6 (4 to 16) | 10.3 (5 to 11) | 6.5 (4 to 8) |
|  | Personalized approach | 2.2 (1 to 5) | 2.1 (1 to 4) | 3.0 (1 to 4) |

Note: The numbers in parentheses are the empirical 95%CI.
