# Supplementary material for: Revisiting the guidelines for ending isolation for COVID-19 patients
Source: eLife. 2021 Jul 27;10:e69340. doi: 10.7554/eLife.69340 (PMC8315804; doi:10.7554/eLife.69340)
Supplement: Supplementary file 1. [file elife-69340-supp1.docx]

**Supplementary File 1. Estimated parameters of the three models**

| Parameter Name | Symbol (Unit) | Baseline model | “Eclipse phase” model | “Innate immune response” model |
| --- | --- | --- | --- | --- |
| Maximum rate constant for viral replication | $\gamma$ (day^-1^) | $3.80$ | $2.87\times{10}^{6 \&}$ | $6.98$ |
| Rate constant for virus infection | $\beta$ ([copies/ml]^-1^ day^-1^) | $7.9\times{10}^{-6}$ | $4.8\times{10}^{-6}$ | $1.6\times{10}^{-6}$ |
| Death rate of infected cells | $\delta$ (day^-1^) | $0.68$ | $0.84$ | $2.10$ |
| Viral load at symptom onset | $V(0)$ (copies/ml) | $3.3\times{10}^{4}$ | $5.2\times{10}^{4}$ | $393\times{10}^{4}$ |
| Ratio of infected cells | $f_{I}\left( 0 \right)$ (unitless) | -- | ${0.1}^{\#}$ | -- |
| Mean length of eclipse phase | $1/k$ (day) | -- | ${1/3}^{*}$ | -- |
| Concentration of INFs | $F\left( 0 \right)$ (concentration) | -- | -- | $8.3\times{10}^{3}$ |
| Concentration of INFs that produces a half-maximum rate constant for viral replication | $1/\eta$ (concentration) | -- | -- | ${1.0\times{10}^{4}}^{\#}$ |
| Rate constant which is proportional to secretion of INFs from infected cells | $s$ (day^-1^) | -- | -- | ${0.01}^{\#}$ |
| Removal rate of IFNs | $\alpha$ (day^-1^) | -- | -- | ${0.7}^{\#}$ |

^&^The unit is [copies/ml] day^-1^. ^*^Parameter values are fixed based on previous studies (Gonçalves et al., 2020; Néant et al., 2021). ^#^Parameter values are assumed.
