# Supplementary material for: Revisiting the guidelines for ending isolation for COVID-19 patients
Source: eLife. 2021 Jul 27;10:e69340. doi: 10.7554/eLife.69340 (PMC8315804; doi:10.7554/eLife.69340)
Supplement: Supplementary file 2. [file elife-69340-supp2.docx]

**Supplementary File 2. AIC and BIC of the three models.**

|  | Baseline model | “Eclipse phase” model | “Innate immune response” model |
| --- | --- | --- | --- |
| AIC | 918.9 | 916.1 | 913.9 |
| BIC | 931.5 | 928.7 | 929.3 |
